# DSC Brain Perfusion Using Advanced Deconvolution Models in the Diagnostic Work-Up of Dementia and Mild Cognitive Impairment: A Semiquantitative Comparison with HMPAO-SPECT-Brain Perfusion

**DOI:** 10.3390/jcm9061800

**Published:** 2020-06-09

**Authors:** Manuel A. Schmidt, Tobias Engelhorn, Stefan Lang, Hannes Luecking, Philip Hoelter, Kilian Froehlich, Philipp Ritt, Juan Manuel Maler, Torsten Kuwert, Johannes Kornhuber, Arnd Doerfler

**Affiliations:** 1 Departments of Neuroradiology, Friedrich-Alexander-University Erlangen-Nuremberg, Schwabachanlage 6, 91054 Erlangen, Germany; tobias.engelhorn@uk-erlangen.de (T.E.); S.Lang@uk-erlangen.de (S.L.); hannes.luecking@uk-erlangen.de (H.L.); philip.hoelter@uk-erlangen.de (P.H.); arnd.doerfler@uk-erlangen.de (A.D.); 2 Departments of Neurology, Friedrich-Alexander-University Erlangen-Nuremberg, Schwabachanlage 6, 91054 Erlangen, Germany; kilian.froehlich@uk-erlangen.de; 3 Department of Nuclear Medicine, Friedrich-Alexander-University Erlangen-Nuremberg, Ulmenweg 18, 91054 Erlangen, Germany; philipp.ritt@uk-erlangen.de (P.R.); torsten.kuwert@uk-erlangen.de (T.K.); 4 Departments of Psychiatry, Friedrich-Alexander-University Erlangen-Nuremberg, Schwabachanlage 6, 91054 Erlangen, Germany; manuel.maler@uk-erlangen.de (J.M.M.); johannes.kornhuber@uk-erlangen.de (J.K.)

**Keywords:** dementia, brain perfusion, DSC (dynamic susceptibility contrast) perfusion, SPECT (single-photon emission-computed tomography)

## Abstract

Background: SPECT (single-photon emission-computed tomography) is used for the detection of hypoperfusion in cognitive impairment and dementia but is not widely available and related to radiation dose exposure. We compared the performance of DSC (dynamic susceptibility contrast) perfusion using semi- and fully adaptive deconvolution models to HMPAO-SPECT (99mTc-hexamethylpropyleneamine oxime-SPECT). Material and Methods: Twenty-seven patients with dementia of different subtypes including frontotemporal dementia (FTD) and mild cognitive impairment (MCI) received a multimodal diagnostic work-up including DSC perfusion at a clinical 3T high-field scanner and HMPAO-SPECT. Nineteen healthy control individuals received DSC perfusion. For calculation of the hemodynamic parameter maps, oscillation-index standard truncated singular value decomposition (oSVD, semi-adaptive) as well as Bayesian parameter estimation (BAY, fully adaptive) were performed. Results: Patients showed decreased cortical perfusion in the left frontal lobe compared to controls (relative cerebral blood volume corrected, rBVc: 0.37 vs. 0.27, *p* = 0.048, adjusted for age and sex). Performance of rBVc (corrected for T1 effects) was highest compared to SPECT for detection of frontal hypoperfusion (sensitivity 83%, specificity 80% for oSVD and BAY, area under curve (AUC) = 0.833 respectively, *p* < 0.05) in FTD and MCI. For nonleakage-corrected rBV and for rBF (relative cerebral blood flow), sensitivity of frontal hypoperfusion was above 80% for oSVD and for BAY (rBV: sensitivity 83%, specificity 75%, AUC = 0.908 for oSVD and 0.917 for BAY, *p* < 0.05 respectively; rBF: sensitivity 83%, specificity 65%, AUC = 0.825, *p* < 0.05 for oSVD). Conclusion: Advanced deconvolution DSC can reliably detect pathological perfusion alterations in FTD and MCI. Hence, this widely accessible technique has the potential to improve the diagnosis of dementia and MCI as part of an interdisciplinary multimodal imaging work-up. Advances in knowledge: Advanced DSC perfusion has a high potential in the work-up of suspected dementia and correlates with SPECT brain perfusion results in dementia and MCI.

## 1. Introduction

Prevalence of dementia is rather high in the elderly population (>60 years) with about 5–7% worldwide [1]. Alzheimer’s disease is the most common cause; other etiologies include frontotemporal dementia (FTD), dementia with Lewy bodies (DLB), vascular dementia (VD) and cortico-basal degeneration (CBD) [2]. Early and reliable diagnosis is important to prevent unnecessary procedures, uncertainty for patients and relatives as well as delay in the initiation of symptomatic treatments [3,4,5]. Structural MRI already plays an important role in the diagnosis of early dementia [6], but perfusion imaging helps identifying subtypes, which is important for therapy decisions and further care of patients [3,7]. 99mTc-hexamethylpropyleneamine oxime single-photon emission-computed tomography (HMPAO-SPECT) is used in the diagnostic work-up as specific regional patterns of hypoperfusion have been described for different subtypes of dementia [8]. However, the radiation dose exposure for head SPECT/CT is reported to be as high as 4.1 mSv on average [9]. This is 41-fold the dose of a single chest X-ray examination. Thus, an alternative imaging modality without radiation exposure is desirable.

Earlier evaluation of the potential of DSC-(dynamic susceptibility contrast)-perfusion as an imaging biomarker of the cerebral microcirculation in AD has been promising [10]. However, correlation analysis of DSC-perfusion with SPECT has produced mixed results in the past. Studies investigating rBV (relative cerebral blood volume) and rBF (relative cerebral blood flow) measures in Alzheimer’s disease have partly shown promising results and good correlation with SPECT [11,12]. Several studies demonstrated that DSC perfusion cannot replace SPECT in AD and that SPECT remains superior over DSC perfusion regarding diagnostic accuracy [13,14].

Advanced perfusion deconvolution models like oSVD (oscillation-index standard truncated singular value decomposition) are far less sensitive for differences in tracer arriving time [15] leading to a more robust estimation of rBF and possibly to a superior detection of hypoperfusion in dementia. Another model, Bayesian parameter estimation, a probabilistic method that is considered to deliver even more accurate and robust hemodynamic parameters [16,17], is also a promising technical approach to perfusion imaging in dementia.

These technical improvements as well as higher field strength, higher spatial resolution and advanced deconvolution models for calculation of hemodynamic parameter maps led us to reevaluate DSC perfusion compared to SPECT in early dementia.

## 2. Material and Methods

### 2.1. Patients

Our retrospective study comprised a total of 27 patients with clinically suspected dementia (mean age 66 years ± 9.4 years). Suspected diagnosis included MCI (*n* = 9), FTD (*n* = 7), AD (*n* = 6), DLB (*n* = 2), VD (*n* = 2) CBD (*n* = 1). Patients’ clinical characteristics are summarized in Table 1. Informed consent was obtained from all subjects. The Clinical Investigation Ethics Committee of the University of Erlangen-Nuremberg approved the study protocol on 27 January 2016 (ethical code number 231_12Az) and the research was conducted in accordance with the Declaration of Helsinki. All patients underwent a standardized clinical assessment including a mini mental status test (MMST) and received HMPAO-SPECT as well as DSC perfusion. Additionally, we included a group of 19 healthy control individuals (mean age 57 years ± 8.5 years) with no signs of cognitive impairment. Controls received DSC perfusion only.

### 2.2. Imaging Protocol

DSC perfusion was performed with a clinical high-field scanner at a magnetic field strength of 3T (Magnetom Trio, Siemens, Erlangen, Germany) with a single-shot EPI-(echo planar imaging) -gradient echo sequence (TE (echo time) = 32 ms, TR (repitition time) = 1840 ms, field of view = 230 × 230 mm, slice thickness = 6 mm, slice gap = 1.8 mm, number of slices = 19, matrix = 128 × 128). We used 1 mmol/mL gadubotrol (Gadovist, Bayer Healthcare, Leverkusen, Germany) as a contrast agent. Dose was weight-dependent, 1 mmol/kg, injected as intravenous bolus in the right (preferably) or left cubital vein using an MR-compatible injector (Medtron, Saarbrücken, Germany) at a flow-rate of 5 mL/sec followed by a saline flush of 30 mL (0.9% NaCl). Additionally, anatomical datasets were acquired (3D T2 SPACE dark fluid: TE = 388 ms, TR= 5000 ms, TI (inversion time) = 1800 ms, field of view = 256 × 256 mm, slice thickness = 1 mm isotropic, matrix = 256 × 258 and 3D T1 MPRAGE: TE = 2.52 ms, TR = 1900 ms, TI = 900 ms, field of view = 256 × 256 mm, slice thickness = 1 mm isotropic, matrix = 256 × 256).

A multichannel head coil (32 channels, Siemens, Erlangen, Germany) was used for signal reception.

SPECT/CT acquisitions were carried out as follows: After ten minutes of resting in a quiet surrounding and dimmed light, patients were injected with Tc-99m-HMPAO (Stabilised Ceretec™, GE Healthcare, Munich, Germany). Injected activity was determined by measuring the initially prepared as well as the residual activity in the syringe with proper decay correction. An average of 546 ± 48 MBq (95% CI = 428–637) were injected. The SPECT/CT images were acquired 30 min p.i., using a dual-headed gamma camera (Symbia T2/T6, Siemens Healthcare, Hoffman Estates, IL, USA). The following acquisition parameters were used: low-energy high-resolution collimators, zoom factor 1.23, head-contouring orbit with 60 stops (120 projections) over 360°, acquisition time of 30 s per stop, and 140 keV photopeak window with 15% width. After SPECT, a low-dose CT was acquired for anatomical co-registration and attenuation correction. The images were reconstructed in a 128 × 128 matrix (3.9 mm pixel size) using the OSEM (ordered subset expectation maximization) algorithm (Flash3D). Post-reconstruction smoothing of images was carried out using a Gaussian filter with 6.0 mm FWHM (full width at half maximum). All images were corrected for scattered and attenuated photons using the dual energy window method and the low-dose CT images, respectively.

### 2.3. Image Processing and Analysis

DSC data were transferred to an external workstation for processing with an FDA-(food and drug administration)-approved software package (Olea Sphere, La Ciotat, France). Background segmentation of data sets was adjusted to remove extracranial tissue. The automatic arterial pixel selection tool implemented in the software and based on cluster analysis was chosen for computing an arterial input function (AIF) [18]. Hemodynamic parameter maps of rBV, rBF and MTT (mean transit time) were calculated. rBV parametric maps were calculated with and without correction for contrast agent leakage (rBV and rBVc). We used two different deconvolution models: semi-adaptive, delay insensitive oSVD [15], a further developed model based on nonadaptive sSVD [19], and with the same presets, Bayesian hemodynamic parameter estimation (BAY), which is considered to lead to a more reliable and accurate estimation of perfusion indices [16,17,20]. Bayesian parameter estimation is a probabilistic method that is considered to deliver even more accurate and robust hemodynamic parameters. Compared with previously reported singular value decomposition algorithms, Bayesian analysis of DSC perfusion has been reported to provide better qualitative and quantitative assessments of rBF [21]. Circular ROIs (regions of interest) were placed in the cortex and in deep white matter of the frontal lobe, the parieto-occipital lobe as well as the temporal lobe. Coordinates in MNI space are given in Table 2.

**Figure 1:**
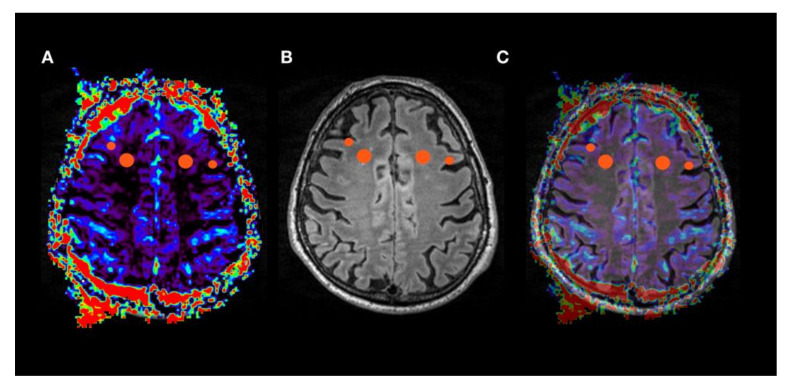
ROIs in rBV (relative cerebral blood volume) map (oSVD - oscillation-index standard truncated singular value decomposition). Circular ROIs were placed in cortex and white matter in frontal lobe (**A**–**C**), as well as in parietal and temporal lobe and in hippocampus in the respective hemodynamic parameter maps rBV (**A**), rBVc (relative cerebral blood volume corrected), rBF (relative cerebral blood flow) and MTT (mean transit time) both for oSVD and Bayesian deconvolution. Data sets were coregistered with an anatomical dataset (**B**, T2 SPACE) and fused (**C**) for correct placement of ROIs.

ROI area was defined as 3 for cortex and 5 for deep white matter in a software inherent arbitrary unit (mean surface for cortex ROI = 25.9 mm^2^, for deep white matter ROI = 80.7 mm^2^). For the exact anatomical placement of ROIs, parametric perfusion maps were co-registered and fused with a 3D T2 SPACE dataset (Figure 1). ROI placement was performed by two neuroradiologists well trained in perfusion MRI who were blinded to the patients’ diagnosis. The ratio of deep white matter/cortical perfusion was calculated for every anatomical localization and for both deconvolution models, thus using white matter as a reference region. The same cortical areas were selected for all subjects.

Regarding evaluation of SPECT images, calculated rBF parametric maps were read by a physician with extensive experience in nuclear medicine and SPECT imaging and visually classified according to frontal, parieto-occipital and temporal hypoperfusion.

### 2.4. Statistical Analysis

Differences in white matter/cortical perfusion ratios between patients and controls were assessed by unpaired Student’s *t*-tests after verification of normal distribution of the data. Additionally, we corrected for age and sex after principal component analysis and adding them as a combined covariate in a general linear model (ANCOVA).

Classification of actual hypoperfusion in SPECT imaging was considered as standard and the performance of the perfusion ratio deep white matter/cortex of every single DSC parameter, i.e., the potential to predict hypoperfusion in SPECT, was tested using receiver-operating characteristics (ROC) with estimation of the AUC (area under the curve). *p* < 0.05 was considered statistically significant. The p value is given under the null hypothesis that the area under the curve equals 0.5. Statistical analysis was performed using SPSS version 19 (IBM, Ehningen, Germany).

## 3. Results

**Figure 2:**
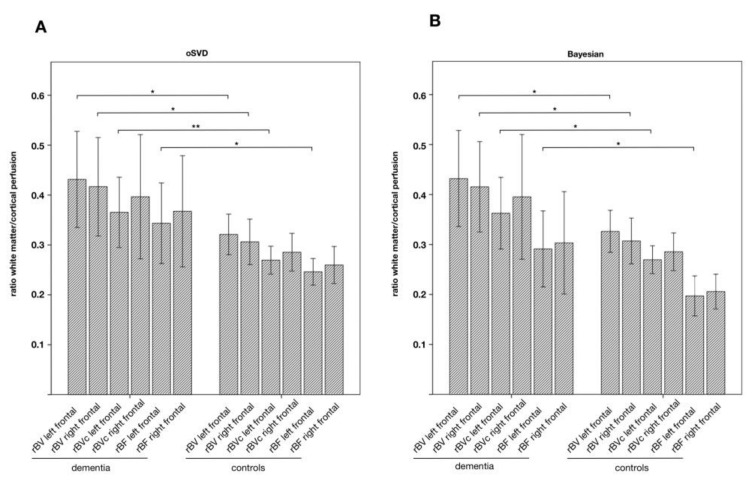
Ratios of white matter/cortical perfusion of the frontal ROIs for both (**A**) oSVD and (**B**) Bayesian deconvolution models in patients with suspected dementia compared to healthy controls. Increased ratio in dementia indicates relative cortical hypoperfusion. Significant results (*p* < 0.05) are marked with *, values that remained significant after correction for age and sex are marked with **.

We found a significantly increased ratio of white matter/cortical perfusion, i.e., relative cortical hypoperfusion, measured with DSC in dementia compared to controls for frontal ROIs (Figure 2).

After correction of the covariates age and sex, the difference in means regarding the rBVc parameter of the left frontal ROI remained significant (0.37 vs. 0.27, *p* = 0.048) whereas it failed to reach statistical significance regarding rBV and rBF for both, the oSVD and the Bayesian approach.

We also looked at the uncorrected values. The ratios of the bilateral frontal lobe for rBV (left: 0.43 vs. 0.32, *p* = 0.037; right: 0.42 vs. 0.31, *p* = 0.043) and of the left frontal lobe for rBF (0.34 vs. 0.25, *p* = 0.025) were significantly higher compared to controls, showing at least a tendency. Regarding Bayesian estimation of parametric perfusion maps, we found similar results for rBV of the bilateral frontal lobe (left: 0.43 vs. 0.33, *p* = 0.045; right 0.42 vs. 0.31; *p* = 0.034), for rBVc of the left frontal lobe (0.36 vs. 0.27, *p* = 0.018) and for rBF of the left frontal lobe (0.29 vs. 0.20, *p* = 0.029).

Concerning the parietal ROIs, cortical hypoperfusion failed to reach statistical significance. MTT was not significantly different regarding the frontal and parietal ROIs. No significant hypoperfusion was found for temporal ROIs with ether the oSVD or the BAY approach. Results are summarized in Table 3.

**Figure 3:**
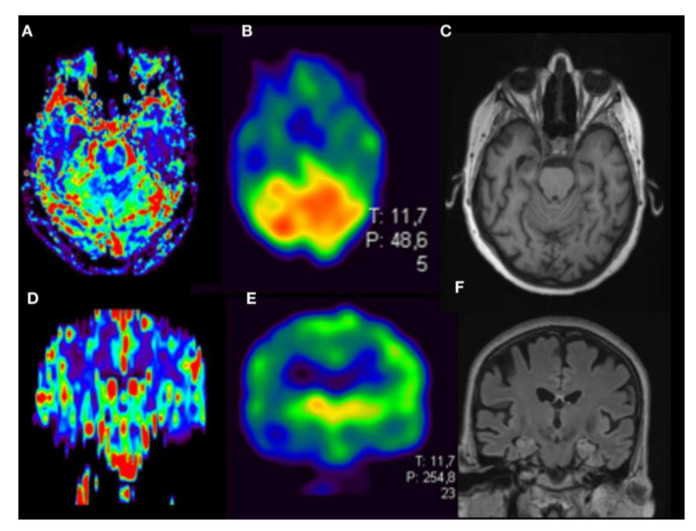
Female with Alzheimer’s disease, 54 y, Mini-Mental-Status-Test score 10. DSC Perfusion (**A**, axial orientation; **D**, coronal orientation rCBVc map) shows typical pattern of frontotemporal and parietal cortical hypoperfusion. Correlation with HMPAO-SPECT. (**B**,**E**) and anatomical datasets with widening of the temporal horns and apico-frontal cisterns as signs of atrophy (T1w in **C**, T2 SPACE in **F**).

**Figure 4:**
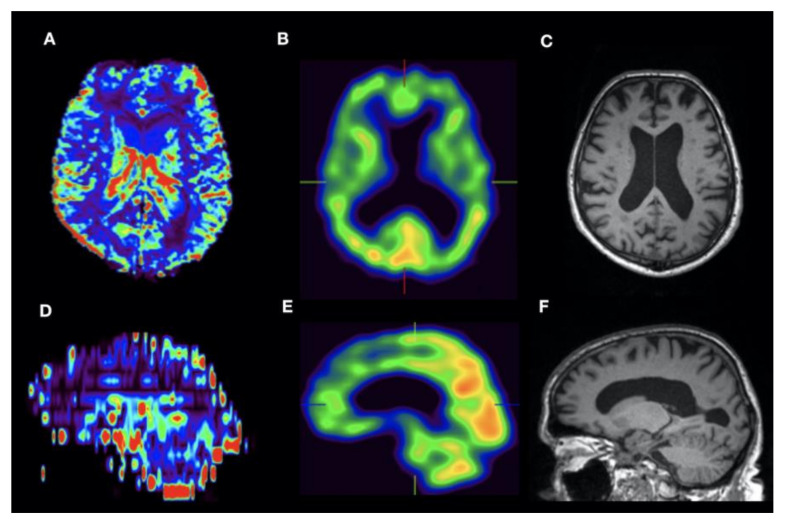
Male with FTD, 69 y, Mini-Mental-Status-Test score 30. DSC Perfusion (**A**, axial orientation; **D**, sagittal orientation rCBVc map) shows typical pattern of frontal cortical hypoperfusion. Correlation with HMPAO-SPECT (**B**,**E**) and the anatomical dataset with ventricular widening as sign of atrophy (T1w in **C** and **F**).

**Figure 5:**
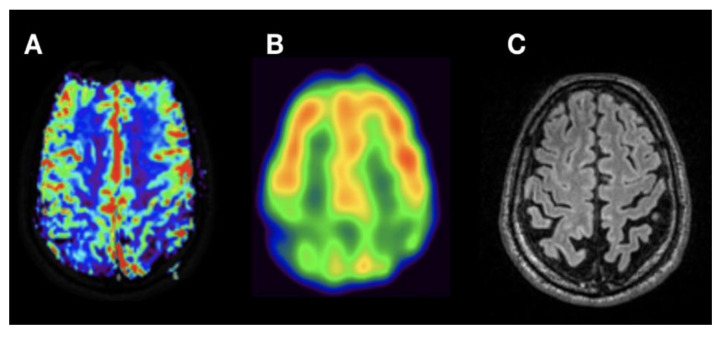
Male with DLB, 60 y, Mini-Mental-Status-Test score 30. DSC Perfusion (**A**, axial orientation rCBVc) shows typical pattern of parieto-occipital cortical hypoperfusion. Correlation with HMPAO-SPECT (**B**) and the anatomical dataset with widening of the parieto-occipital subarachnoid spaces as sign of atrophy (**C**, T2 SPACE).

By visual interpretation of DSC perfusion maps, there was a good correlation of reduced cortical rBVc of temporal and parietal lobe with hypoperfusion seen on the respective SPECT dataset in Alzheimer’s disease (pathological MMST score of 10, Figure 3). Frontal cortical hypoperfusion in DSC correlated with hypoperfusion in SPECT in suspected early FTD in a 69-year-old male (Figure 4). Furthermore, parieto-occipital hypoperfusion in DSC in a 60-year-old male with suspected DLB-matched SPECT hypoperfusion (Figure 5) to a great extent. In this patient, absence of temporomesial atrophy in coronal FLAIR imaging corroborated the suspected diagnosis over posterior cortical atrophy as a variant of AD. Overall, regardless of the clinical diagnosis, there was a high correlation of cortical hypoperfusion in frontal lobes when comparing DSC perfusion with SPECT.

**Figure 6:**
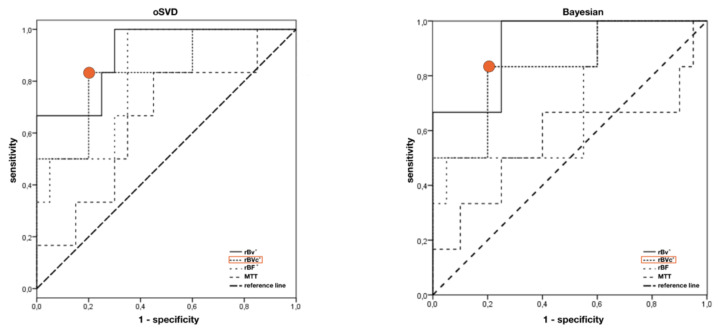
Optimal cut-off points for the ratio of cortical/white matter perfusion in the left frontal lobe for calculated hemodynamic parameter maps for oSVD and BAY deconvolution. rBVc ratio cut off = 0.36 marked red; greater values “predict” hypoperfusion in SPECT with a sensitivity of 83% and specificity of 80%. Significant results (*p* < 0.05) are marked with *.

Using ROC analysis, the sensitivity for predicting hypoperfusion in the left frontal lobe in SPECT was 83% for rBV, rBVc, and rBF both for oSVD and Bayesian deconvolution (Figure 6) at a specificity of 75% for rBV (oSVD and BAY), 80% for rBVc (oSVD and BAY), 65% for rBF (oSVD) and 45% for rBF (BAY) with an AUC above 0.8, respectively (*p* < 0.05). Regarding rBF, only oSVD reached statistical significance (oSVD: AUC = 0.825, *p* < 0.05; BAY: AUC = 0.708, *p* > 0.05). AUC of MTT was not significant for both oSVD and BAY. For the right frontal lobe, as well as for the parietal and the temporal lobe, AUCs did also not reach statistical significance. Results are summarized in Table 4.

## 4. Discussion

DSC perfusion showed cortical hypoperfusion in frontal lobes for both deconvolution models in patients with suspected dementia compared to healthy individuals. The potential of MR-perfusion to detect dementia-induced hypoperfusion in FTD has been shown regarding the arterial spin labeling (ASL) technique, even in early stages of the disease [22,23]. Concerning the composition of our patient group with a majority of patients suspected to develop FTD (7/27) and 2 patients showing signs of an advanced stage of the disease (MMST < 20), our results are in line with these reports. Parieto-occipital cortical hypoperfusion failed to reach statistical significance in our cohort, presumably because of the relatively small sample size of patients with this pattern of hypoperfusion. We could not show cortical hypoperfusion in temporal lobes, although this has been described [23]. This may be due to partial volume effects and susceptibility artifacts near the skull base and air-containing structures.

An age-dependency of brain perfusion parameters has been described [24]. In an extensive and thorough analysis of 175 consecutive scans, Shin et al. report a decrease of rBV of 3.7% per decade in grey matter. Similar decrease rates of CBF and increase rates of MTT per decade are reported. Our results indicate a much stronger effect with a 26% difference of rBV and rBF regarding patients vs. controls. Given the age difference of 9 years between patients and controls, this strong effect cannot be explained as age-effect alone, as one would expect roughly 5% difference of rBV that could be addressed to aging. Additionally, it has been reported that brain perfusion is not homogeneously decreased in the ageing brain but is characterized by decreased rBF in frontal and parietal areas and cerebellum, and relatively preserved in temporal, occipital and orbital frontal areas. Moreover, it has been suggested that observed differences in brain perfusion can also be ascribed to changes in brain volume—at least to a certain extent [25]. Our finding of cortical hypoperfusion remained significant for the left frontal ROI of the rBVc parameter after adjustment of age and sex.

Correlation analysis of DSC perfusion with SPECT has produced mixed results in the past. Studies investigating rBV and rBF measures in Alzheimer’s disease have partly shown promising results and good correlation with SPECT [11,12]. However, several studies demonstrated that DSC perfusion cannot replace SPECT in AD and that SPECT remains superior over DSC perfusion regarding diagnostic accuracy [13,14].

Nevertheless, we show a good visual correlation of cerebral blood volume derived from DSC with SPECT brain perfusion maps in AD (Figure 3). Temporal and parietal hypoperfusion could be clearly delineated on both parametric maps on corresponding slices. In case of temporal hypoperfusion, a potential advantage of DSC perfusion is the higher spatial resolution that can be achieved over SPECT [26,27]. Combination with additional evaluation of anatomical datasets, shows typical temporomesial and frontoparietal atrophy.

Furthermore, we could demonstrate a high correlation of frontal cortical hypoperfusion in rBVc with the respective SPECT rBF image in suspected frontotemporal dementia (Figure 4). As shown, the possibility of multiplanar reconstruction of DSC hemodynamic parameter maps helps in identifying a frontoparietal gradient of cortical perfusion in the color-coded rBVc map when comparing DCS and SPECT datasets.

Moreover, there was good correlation of hypoperfusion in DSC and SPECT in a case of DLB (Figure 5). Although the atrophy pattern of DLB may be very heterogeneous, relative preservation of the medial temporal lobe has been reported [28,29]. Parieto-occipital cortical hypoperfusion can be clearly delineated in the DSC rBVc map and in the SPECT rBF map correlating with focal atrophy. Additional coronal imaging shows absence of temporomesial atrophy (Figure 5B).

Focal patterns of hypoperfusion in DSC rBVc maps for AD, FTD and DLB are in line with previously reported patterns of hypoperfusion in SPECT in these etiologies [8].

Quantitative DSC analysis revealed a rBVc white matter/cortex-ratio greater 0.36 can predict visually detectable frontal hypoperfusion in SPECT with a sensitivity of 83% and a specificity of 80%. Absolute quantification of blood volume and blood flow is not possible because they depend on scanner, applied contrast agent and acquisition parameters and can be influenced by partial volume effects. Thus, we have chosen a ratio of white matter/cortical perfusion, using white matter as a reference region, to overcome these issues. These ratios can be easily calculated and transferred into clinical routine. Performance of rBVc was independent of the chosen deconvolution model (Figure 6), as the calculated blood volume is only dependent on the area under the R2* curve [17,30].

Our results are in line with previous reports of a promising correlation of rBV derived from DSC perfusion compared to rBF by SPECT [11]; however, our settings with higher field strength (3T vs. 1.5T), and thus higher signal-to-noise ratio (SNR), and improved spatial resolution (slice thickness 6 mm/interslice gap 1.8 mm vs. 7 mm/3 mm) make data acquisition more robust in terms of reproducibility and accuracy [31]. Interestingly, specificity was slightly higher for rBVc than for uncorrected (i.e., not corrected for contrast agent leakage) rBV even though no disruption of the blood-brain barrier (BBB) was suspected. One possible reason may be subtle BBB disruption as dysfunction of the BBB. This phenomenon has been described in AD before [32] and might also apply for FTD and DLB.

Despite a high performance of rBVc, we could show an exactly as high sensitivity of 83% for rBF to predict hypoperfusion in SPECT. This seems to be in contrast to previous studies where correlation of DSC derived rBF with SPECT was found to be poor [13,14]. However, in these studies, nonadaptive singular value deconvolution was used for deconvolving the tissue signal from the arterial input function. This technique has been described to be sensitive to delay and dispersion of the arriving contrast agent bolus leading to over- or underestimation of cerebral blood flow in tissues where the bolus arrives earlier than in the chosen AIF [15]. Consequently, formerly poor correlation of rBF with SPECT might have been due to the fact that true rBF has been underestimated by the deconvolution algorithm. oSVD, as used in our setting, in contrast is far less sensitive for differences in tracer arriving time [15] leading to a more robust estimation of rBF and a superior detection of hypoperfusion.

When using Bayesian parameter estimation, a probabilistic method that is considered to deliver even more accurate and robust hemodynamic parameters [16,17], AUC of rBF estimation failed to reach statistical significance (Figure 6).

MTT estimations were generally lower when comparing the values calculated with the Bayesian approach to the values calculated with oSVD. It has been described that SVD techniques have larger errors in slow flow conditions and the parameter estimation performance is poorer compared to high flow conditions [15]. Thus, in slow flow conditions such as suspected dementia, MTT estimation with the Bayesian method may be more accurate, despite the values failed to reach statistical significance. Additionally, Bayesian deconvolution has been reported to outperform oSVD, especially in cases of high cerebral blood flow [20]. This seems to be of less relevance in detecting hypoperfusion in dementia with low CBF.

A combination of delay-insensitive oSVD derived rBVc and rBF perfusion indices thus leads to a high performance in predicting hypoperfusion in SPECT brain perfusion in dementia and MCI.

Our study has certain limitations. Firstly, the number of 27 patients included is rather small. Certainly, larger series are needed to confirm the results and also test for diagnostic accuracy of DSC in dementia. Regarding the known age-dependency of brain perfusion, it has to be taken into account that patients and controls are not age-matched in our analysis. However, the results remain significant after adjusting for age and sex effects and it can be assumed that the age-effect is relatively weak compared to the significant differences of brain perfusion parameters that we observed. Another shortcoming is the lack of quantitative data corroborating the visual correlation of DSC and SPECT hypoperfusion patterns; this also necessitates further case series. The heterogeneity of our cohort probably limits general conclusions about the correlation of DSC and SPECT data, despite providing a way to examine various perfusion patterns regarding the suspected clinical diagnosis.

## 5. Conclusions

Advanced oSVD deconvolution in DSC perfusion has a high potential and correlates with SPECT brain perfusion results in dementia and MCI. Hence, DSC perfusion could be helpful in an interdisciplinary diagnostic work-up of suspected dementia.

## Figures and Tables

**Table 1:** Patients’ clinical characteristics.

Patient No	Age	Gender	Suspected Diagnosis	MMST
1	69	f	FTD	27
2	57	m	FTD	27
3	50	m	AD	27
4	79	f	FTD	23
5	55	m	MCI	30
6	83	m	FTD	19
7	70	f	FTD	29
8	78	f	DLB	20
9	53	m	CBD	24
10	63	m	MCI	29
11	54	f	AD	10
12	73	f	DLB	28
13	78	f	MCI	25
14	58	f	MCI	28
15	62	f	FTD	14
16	69	m	FTD	30
17	68	m	AD	26
18	77	m	AD	27
19	56	m	MCI	28
20	58	m	MCI	29
21	73	f	VD	27
22	74	f	MCI	27
23	60	m	AD	30
24	69	m	VD	28
25	54	m	MCI	26
26	63	m	MCI	26
27	69	f	AD	22

f = female, m = male, FTD = frontotemporal dementia, AD = Alzheimer’s disease, MCI = mild cognitive impairment, DLB = dementia with Lewy bodies, CBD = cortico-basal degeneration, VD = vascular dementia; MMST = mini-mental status test score.

**Table 2:** Coordinates of the chosen frontal, parietal and temporal ROIs (regions of interest) in MNI (Montreal Neurological Institute) space.

	MNI Cortex			MNI White Matter		
	X	Y	Z	X	Y	Z
left frontal	−36	2	53	−12	7	51
left parietal	−28	−68	41	−19	−53	41
left temporal	−53	−7	−22	−42	−1	−22
right frontal	24	11	53	17	6	53
right parietal	28	−68	41	19	−53	41
right temporal	53	−7	−22	42	−1	−22

**Table 3:** Ratios of white matter/cortex perfusion values of relative cerebral blood volume (rBV), relative cerebral blood volume corrected for leakage (rBVc), relative cerebral blood flow (rBF) and the value of the cortical ROI of mean transit time (MTT) [ms].

oSVD	Patients				Controls			
	rBV	rBVc	rBF	MTT [ms]	rBV	rBVc	rBF	MTT [ms]
left frontal	0.43 * ± 0.24	0.37 ** ± 0.17	0.34 * ± 0.2	4720 ± 1430	0.32 ± 0.08	0.27 ± 0.06	0.25 ± 0.06	4930 ± 870
right frontal	0.42 * ± 0.24	0.40 ± 0.21	0.37 ± 0.18	4860 ±1520	0.31 ± 0.1	0.29 ± 0.08	0.26 ± 0.08	4920 ± 850
left parietal	0.41 ± 0.18	0.41 ± 0.2	0.40 ± 0.2	5990 ± 3710	0.44 ± 0.14	0.37 ± 0.12	0.36 ± 0.13	4760 ± 750
right parietal	0.52 ± 0.25	0.44 ± 0.28	0.44 ± 0.25	5190 ± 2280	0.43 ± 0.13	0.35 ± 0.1	0.35 ± 0.1	5050 ± 770
left temporal	0.58 ± 0.26	0.49 ± 0.17	0.51 ± 0.15	4810 ± 1930	0.49 ± 0.14	0.46 ± 0.1	0.46 ± 0.12	4880 ± 940
right temporal	0.47 ± 0.21	0.43 ± 0.16	0.44 ± 0.14	5540 ± 2520	0.43 ± 0.17	0.42 ± 0.12	0.39 ± 0.12	5000 ± 1050
**Bayesian**	**patients**				**controls**			
left frontal	0.43 * ± 0.24	0.36* ± 0.18	0.29 * ± 0.19	3300 ± 1320	0.33 ± 0.09	0.27 ± 0.06	0.20 ± 0.08	3290 ± 1680
right frontal	0.42 * ± 0.22	0.40 ± 0.31	0.30 ± 0.15	3940 ± 3150	0.31 ± 0.1	0.29 ± 0.08	0.21 ± 0.07	3370 ± 1240
left parietal	0.42 ± 0.17	0.40 ± 0.2	0.33 ± 0.18	4990 ± 4070	0.44 ± 0.15	0.37 ± 0.12	0.26 ± 0.08	3080 ± 920
right parietal	0.52 ± 0.24	0.44 ± 0.28	0.39 ± 0.31	3690 ± 1980	0.43 ± 0.13	0.35 ± 0.1	0.26 ± 0.06	3320 ± 910
left temporal	0.50 ± 0.21	0.48 ± 0.18	0.44 ± 0.2	3840 ± 1890	0.49 ± 0.15	0.46 ± 0.1	0.40 ± 0.15	3650 ± 1080
right temporal	0.52 ± 0.2	0.42 ± 0.16	0.39 ± 0.17	4440 ± 2450	0.43 ± 0.17	0.42 ± 0.1	0.36 ± 0.13	4250 ± 3060

Patients vs. controls, values given as mean ± standard deviation, significant results (*p* < 0.05) are marked with an *. Values that remained significant after correction for age and sex are marked with **.

**Table 4:** Cut-off ratios of white matter/cortex perfusion values of relative cerebral blood volume (rBV), relative cerebral blood volume corrected for leakage (rBVc), relative cerebral blood flow (rBF) and mean transit time (MTT) calculated by ROC analysis.

	oSVD				Bayesian			
	rBV	rBVc	rBF	MTT	rBV	rBVc	rBF	MTT
**left frontal**								
Cut off ratio	0.4	0.36	0.29	1.18	0.41	0.36	0.21	1.39
Sensitivity	83%	83%	83%	83%	83%	83%	83%	67%
specificity	75%	80%	65%	55%	75%	80%	45%	60%
AUC	0.908 *	0.833 *	0.825 *	0.650	0.917 *	0.833 *	0.708	0.567
**right frontal**								
Cut off ratio	0.33	0.3	0.27	1.18	0.33	0.30	0.21	0.92
Sensitivity	60%	60%	60%	80%	60%	60%	60%	60%
specificity	42.9%	43%	47.6%	47.6%	42.9%	42.9%	47.6%	14.4%
AUC	0.667	0.657	0.667	0.514	0.667	0.657	0.543	0.581
**left parietal**								
Cut off	0.66	0.91	0.87	-	0.66	0.91	0.77	-
Sensitivity	50%	50%	50%	-	50%	50%	50%	-
specificity	95.8%	100%	100%	-	58%	100%	100%	-
AUC	0.479	0.521	0.542	0	0.479	0.521	0.688	0.021
**right parietal**								
Cut off ratio	0.65	0.97	0.91	-	0.67	0.97	0.83	-
Sensitivity	66.7%	66.7%	66.7%	-	66.7%	66.7%	66.7%	-
specificity	82.6%	100%	100%	-	87%%	100%	100%	-
AUC	0.609	0.667	0.667	0.203	0.609	0.681	0.739	0.072
**left temporal**								
Cut off ratio	0.73	0.55	0.85	1.03	0.77	0.55	0.42	1.47
Sensitivity	50%	50%	25%	75%	50%	50%	75%	75%
specificity	77.3%	72.7%	100%	50%	77.3%	72.7%	59.1%	54.5%
AUC	0.591	0.545	0.466	0.568	0.602	0.557	0.625	0.602
**right temporal**								
Cut off ratio	0.47	0.43	0.45	1.03	0.47	0.43	0.35	1.4
Sensitivity	75%	75%	75%	75%	100%	75%	75%	50%
specificity	50%	59.1%	63.6%	36.4%	45.5%	59.1%	54.5%	63.6%
AUC	0.659	0.591	0.716	0.466	0.625	0.602	0.648	0.432

AUC = area under the curve, significant results (*p* < 0.05) are marked with *.
